# Long-term bleeding events post-percutaneous coronary intervention in patients with malignancy with and without anticoagulant therapy

**DOI:** 10.1007/s12928-025-01151-4

**Published:** 2025-06-09

**Authors:** Yasuhiro Otsuka, Masanobu Ishii, So Ikebe, Tatsuya Tokai, Taishi Nakamura, Kenichi Tsujita, Naoyuki Akashi, Hideo Fujita, Yasuhiro Nakano, Tetsuya Matoba, Takahide Kohro, Yusuke Oba, Tomoyuki Kabutoya, Kazuomi Kario, Yasushi Imai, Arihiro Kiyosue, Yoshiko Mizuno, Kotaro Nochioka, Masaharu Nakayama, Takamasa Iwai, Yoshihiro Miyamoto, Hisahiko Sato, Ryozo Nagai, the CLIDAS research group

**Affiliations:** 1 Department of Cardiovascular Medicine, Graduate School of Medical Sciences, Kumamoto University, Kumamoto, Japan; 2 Department of Medical Information Science, Graduate School of Medical Sciences, Kumamoto University, 1-1-1 Honjo Chuo-Ku, Kumamoto, 860-8556 Japan; 3 Department of Cardiovascular Medicine, Miyazaki Prefectural Nobeoka Hospital, Miyazaki, Japan; 4 Division of Cardiovascular Medicine, Saitama Medical Center, Jichi Medical University, Saitama, Japan; 5 Department of Cardiovascular Medicine, Kyushu University Graduate School of Medical Sciences, Fukuoka, Japan; 6 Department of Clinical Informatics, Jichi Medical University School of Medicine, Tochigi, Japan; 7 Division of Cardiovascular Medicine, Jichi Medical University School of Medicine, Tochigi, Japan; 8 Division of Clinical Pharmacology, Department of Pharmacology, Jichi Medical University, Tochigi, Japan; 9 Department of Cardiovascular Medicine, the University of Tokyo Hospital, Tokyo, Japan; 10 Development Bank of Japan Inc., Tokyo, Japan; 11 Division of Cardiovascular Medicine, Tohoku University Hospital, Miyagi, Japan; 12 Department of Medical Informatics, Tohoku University Graduate School of Medicine, Miyagi, Japan; 13 Department of Cardiovascular Medicine, National Cerebral and Cardiovascular Center, Osaka, Japan; 14 Open Innovation Center, National Cerebral and Cardiovascular Center, Osaka, Japan; 15 Precision Inc., Tokyo, Japan; 16 Jichi Medical University School of Medicine, Tochigi, Japan

**Keywords:** Malignancy, High bleeding risk, PCI

## Abstract

The prevalence of malignancies in patients undergoing percutaneous coronary intervention (PCI) is increasing with aging. Active malignancy is a significant contributor to high bleeding risk. For cancer patients requiring oral anticoagulant (OAC) therapy, the choice between direct oral anticoagulants (DOAC) and warfarin is critical. The aim of this study was to investigate long-term bleeding events in patients with malignancy undergoing PCI. The CLIDAS (Clinical Deep Data Accumulation System) multicenter database includes data from seven tertiary medical hospitals in Japan. This retrospective analysis included 6451 patients who underwent PCI between April 2013 and March 2019 and completed 3-year follow-up. The patients were divided into two groups; No malignancy (n = 5787) and Malignancy group (n = 664). Malignancy was defined by a history of cancer treatment. These groups were further subcategorized based on OAC therapy; (1) No malignancy without OAC (n = 5134), (2) No malignancy with DOAC (n = 261), (3) No malignancy with warfarin (n = 392), (4) Malignancy without OAC (n = 589), (5) Malignancy with DOAC (n = 38), and (6) Malignancy with warfarin (n = 37). The primary outcome was the incidence of bleeding events, defined according to the Global Use of Streptokinase and t-PA for Occluded Coronary Arteries classification of moderate and severe bleeding. The secondary outcomes were major adverse cardiac events (MACE) and net adverse clinical events (NACE). Multivariable Cox regression analysis showed that the malignancy with warfarin group had a significantly higher risk of bleeding events compared to the malignancy without OAC group (hazard ratio [HR], 3.64; 95% confidence interval [CI], 1.38–9.61, p value = 0.009). No significant differences were observed for MACE (HR, 1.39; 95% CI 0.59–3.25, p value = 0.454) or NACE (HR, 1.62; 95% CI, 0.80–3.29; p value = 0.184). Malignancy patients receiving warfarin were associated with a higher risk of bleeding events. DOACs may represent a preferable alternative to warfarin with regard to bleeding risk in patients with malignancy undergoing PCI.

**Graphical abstract:**

Patients with malignancy receiving warfarin after PCI experienced a higher incidence of bleeding events. An analysis of 6,451 patients who underwent PCI revealed that only the group of patients with malignancy on warfarin therapy had a significantly higher incidence of bleeding events over the three-year postoperative period. PCI, percutaneous coronary intervention; OAC, oral anticoagulants; DOAC, direct oral anticoagulants; WF, warfarin.

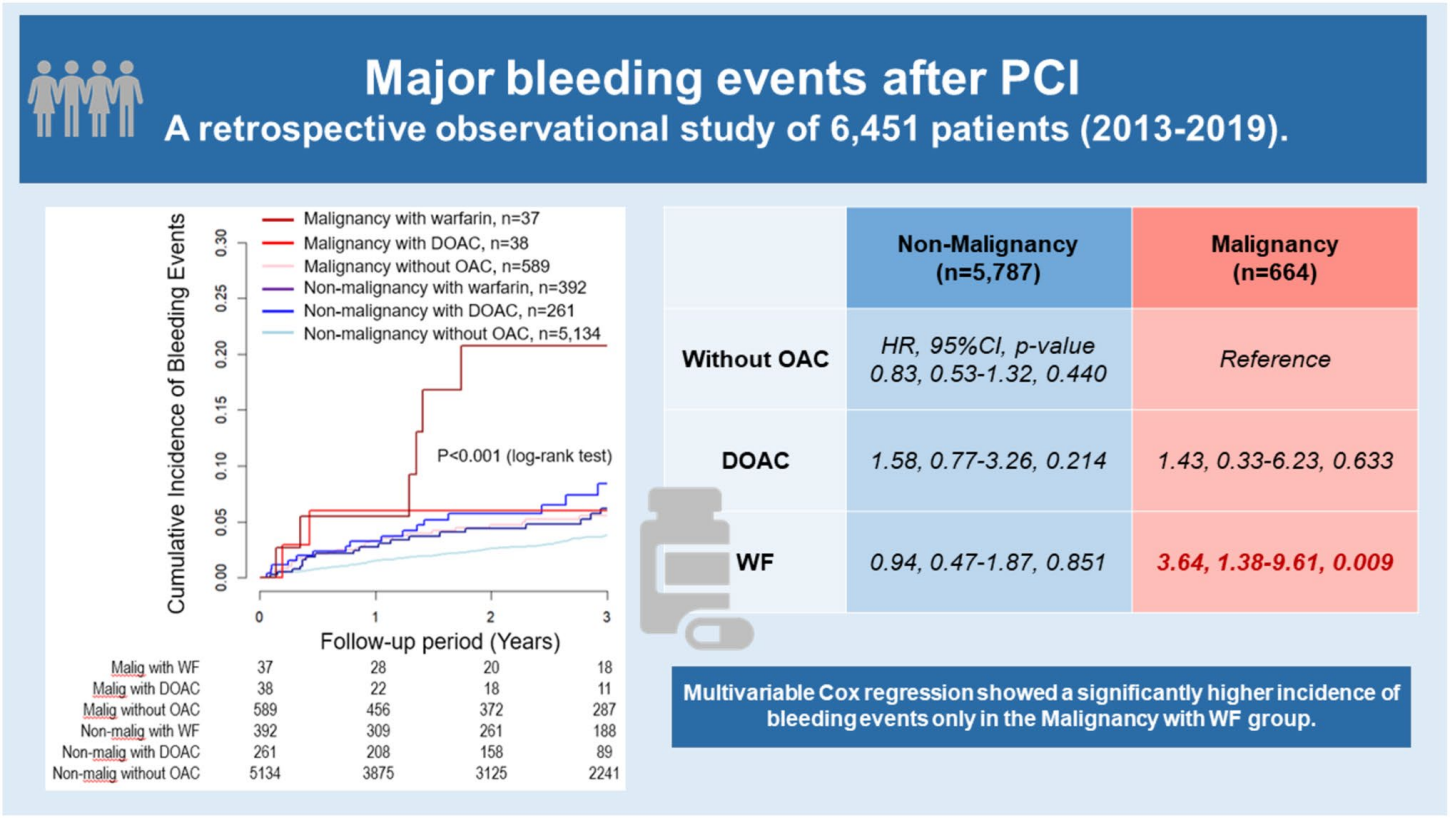

**Supplementary Information:**

The online version contains supplementary material available at 10.1007/s12928-025-01151-4.

## Introduction

Dual antiplatelet therapy (DAPT) is the standard treatment for ischemic heart disease (IHD) following percutaneous coronary intervention (PCI). However, prolonged DAPT use significantly increases the risk of hemorrhagic complications [1]. Severe bleeding is strongly associated with subsequent major adverse cardiac and cerebrovascular events [2]. Therefore, determining the appropriate antithrombotic therapy, including the optimal duration of DAPT, is essential by carefully assessing the patient’s bleeding risk. The Academic Research Consortium (ARC) has established criteria for defining high bleeding risk (HBR) in patients undergoing PCI, known as the ARC-HBR [3]. In Japan, the Japan-HBR adds heart failure, chronic kidney disease, frailty, and peripheral artery disease (PAD) to the ARC-HBR criteria and is widely used [4]. Both ARC-HBR and Japan-HBR identify active malignancy within the past 12 months (i.e., those diagnosed and/or requiring treatment within the past 12 months) as a significant risk factor for HBR. Previous study reported that 3–9% of patients with IHD have a history of malignancy, and it is expected that the coexistence of these conditions will become more common in clinical practice [5, 6]. When oral anticoagulant (OAC) therapy is required after PCI, careful consideration of HBR is crucial, particularly in selecting between direct oral anticoagulants (DOAC) and warfarin (WF) in patients with malignancy. The aim of this study was to investigate long-term bleeding events in patients with malignancy undergoing PCI with and without OAC therapy.

## Methods

### Database

The Clinical Deep Data Accumulation System (CLIDAS) was a multicenter database with seven tertiary medical hospitals in Japan, and was developed from the Japan Ischemic Heart Disease Multimodal Prospective Data Acquisition for preCision Treatment project and aimed to build a clinical data registry system that electronically collects medical records and multimodal data, including coronary angiography and PCI report [7–9]. It collected data from electronic medical records on patients characteristics, oral medicine, injectable drugs, laboratory tests, physiological tests, echocardiographic parameters, electrocardiograms, cardiac catheterizations, and PCI treatment using Standardized Structured Medical Information eXchange version 2 standard and extended storages. Data on patient background information and long-term prognosis were collected by each hospital data manager [10–12]. After anonymization, each institutions’ data was sent through its own multi-purpose clinical data repository system server to the CLIDAS server. Finally, each researcher can analyze the data stored on the CLIDAS server. This study is registered at UMIN (University Hospital Medical Information Network) clinical Trial Registry with registration identification number UMIN000057338.

### Study population

**Fig. 1:**
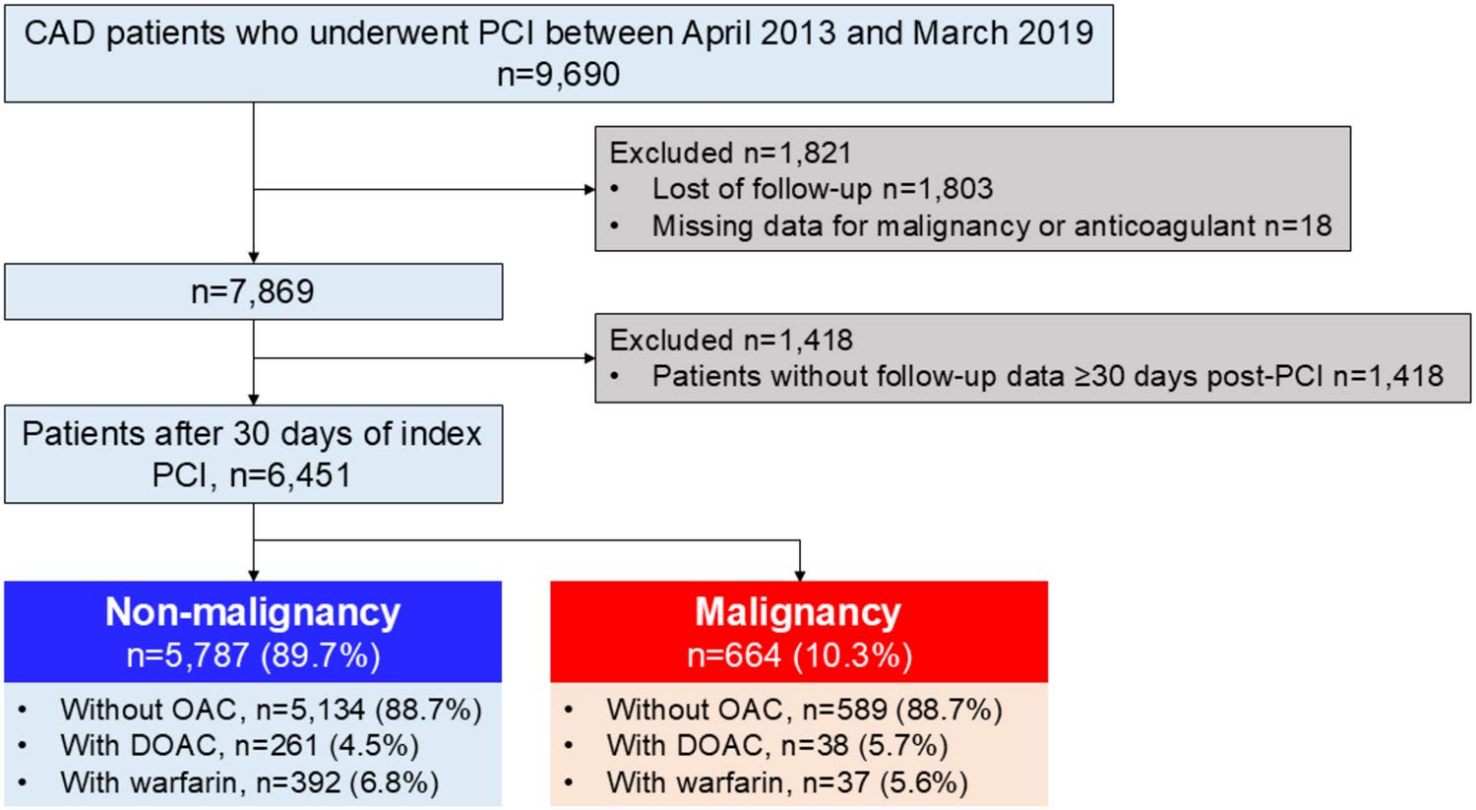
Study flow chart

A total of 9690 consecutive patients admitted for diagnosis or treatment of IHD at a CLIDAS-accredited facilities and undergoing PCI between April 2013 and March 2019 were screened. After excluding 1,418 patients due to missing data on malignancy or anticoagulant use, loss to follow-up, or those undergoing PCI within 30 days of the index procedure, 6,451 patients were enrolled. The patients were divided into two groups: No Malignancy (n = 5787) and Malignancy (n = 664), defined as a history of cancer treatment. These groups were further subdivided based on use of OAC into six categories: *(1)* No Malignancy without OAC (n = 5,134), *(2)* No Malignancy with DOAC (n = 261),* (3)* No Malignancy with WF (n = 392),* (4)* Malignancy without OAC (n = 589),* (5)* Malignancy with DOAC (n = 38) and *(6)* Malignancy with WF (n = 37) (Fig. 1). Regarding the choice of anticoagulant agent, the decision to prescribe either a DOAC or warfarin was made at the discretion of the attending physicians at each participating institution. This decision was based on several clinical factors, including the presence of renal dysfunction, bleeding risk, and the underlying indication for anticoagulation therapy, such as atrial fibrillation, mechanical heart valves, or a history of thromboembolic events (e.g., cerebral infarction).

### Variables

Brain Natriuretic Peptide (BNP) levels were obtained from the lowest value recorded within 30 days before and after the index PCI. Baseline laboratory data were averaged from measurements taken 60 days before to 30 days after the index PCI. Hypertension was defined as systolic blood pressure ≥ 140 mmHg, diastolic blood pressure ≥ 90 mmHg, or ongoing antihypertensive treatment [13]. Diabetes mellitus (DM) was defined as a hemoglobin A1C level ≥ 6.5%, casual blood glucose level ≥ 200 mg/dL, fasting blood glucose level ≥ 126 mg/dL, or ongoing DM treatment [14]. Dyslipidemia was defined as ongoing treatment for dyslipidemia at the time of PCI. The estimated glomerular filtration rate (eGFR) was calculated using the following formula: eGFR = 194 × Cr^−1.094^ × age^−0.287^ (men) and eGFR = 194 × Cr^−1.094^ × age^−0.287^ × 0.739 (women) [15]. Chronic kidney disease (CKD) was defined as eGFR < 60 mL/min per 1.73 m^2^ [15]. Hyperuricemia was defined as serum uric acid levels ≥ 7.0 in men and ≥ 6.0 mg/dL in women, or taking urate-lowering drugs, according to previous studies [16]. Echocardiographic data closest to the index PCI, performed withing 100 days before the procedure, were used to calculate left ventricular ejection fraction (LVEF) using the modified Simpson’s rule [17]. The Teichholz method was used for LVEF measurement if the data of the modified Simpson’s rule were unavailable [17]. The number of diseased vessels was defined as coronary arteries with ≥ 75% stenosis in the major epicardial coronary segments of the right coronary, left anterior descending, and left circumflex arteries or their branches that underwent PCI. The diseased left main trunk (LMT) was defined as ≥ 75% stenosis and counted separately. The patients were categorized according to the combination of the number of diseased vessels and LMT disease.

### Clinical outcomes

The primary outcome was the incidence of major bleeding events, classified as moderate or severe bleeding according to the Global Use of Streptokinase and t-PA for Occluded Coronary Arteries (GSUTO) bleeding criteria [18]. Moderate bleeding requires blood transfusion without hemodynamic compromise, while severe bleeding indicates intracerebral hemorrhage or hemodynamic compromise requiring treatment. Secondary outcomes were major adverse cardiac events (MACE), defined as cardiac death, myocardial infarction (MI), and stroke, as well as net adverse clinical events (NACE), defined as MACE, all-cause death, and bleeding events.

### Statistical analyses

Continuous variables are presented as median values (interquartile ranges) and categorical variables as frequencies and percentages. Group comparisons were analyzed using the Mann–Whitney U test for continuous variables between the two groups, the Kruskal–Wallis test for continuous variables followed by multiple comparisons with the Bonferroni method among the six groups, the chi-squared or Fisher’s exact test for categorical variables. The log-rank test was used for bleeding event comparisons. Cox proportional hazards regression analysis was performed to compute hazard ratios (HRs) and 95% confidence intervals (CIs) as estimates of the clinical outcomes. Multivariable Cox proportional hazard models were adjusted for age, sex, hypertension, DM, dyslipidemia, PAD, atrial fibrillation, CKD, hemodialysis, previous myocardial infarction, prior heart failure hospitalization, previous stroke, acute coronary syndrome (ACS), systolic blood pressure at admission, weight, mean hemoglobin, mean platelet count, and mean BNP levels. The NACE and MACE models also adjusted for previous coronary artery bypass grafting, previous PCI, LMT disease, and multivessel disease. For sensitivity analysis, multiple imputation was performed with 20 imputed datasets generated by the fully conditional specification method, with results combined according to Rubin’s rules [19]. A two-tailed p value < 0.05 denoted a statistically significant. All statistical analyses were performed using R software version 4.0.5 (R Foundation for Statistical Computing, Vienna, Austria; https://www.R-project.org/).

## Results

### Baseline characteristics

The study population of 6,451 patients was divided into No Malignancy and Malignancy groups. The baseline patient characteristics are summarized in Table 1. Compared to the No Malignancy group, patients in the Malignancy group were older and had higher rates of male, CKD, prior heart failure hospitalization, previous stroke, culprit vessel of LMT, higher BNP, and higher serum creatinine levels. In contrast, the patients with Malignancy group had lower body weight, BMI, ACS, systolic and diastolic blood pressure at administration, dyslipidemia, culprit vessel of right coronary artery, hemoglobin (Hgb), platelet count, P2Y12 inhibitor, DAPT, angiotensin converting enzyme inhibitor /angiotensin receptor blocker, β-blocker, statin, Ezetimibe, and proton pump inhibitor.

**Table 1:** Baseline patient characteristics related to medical history and medications

Variables	Missing, %	Overall, N = 6451	Non-malignancy N = 5787	Malignancy N = 664	SMD	p-value
Age, years		71 (64, 78)	71 (63, 78)	75 (70, 80)	− 0.51	< 0.001
Male, n (%)		5,065 (79%)	4,507 (78%)	558 (84%)	− 0.16	< 0.001
Body weight, kg	1.30	63 (56, 71)	64 (56, 72)	61 (54, 69)	0.24	< 0.001
BMI, kg/m2	2.20	24.0 (21.8, 26.4)	24.0 (21.9, 26.5)	23.1 (21.1, 25.7)	0.26	< 0.001
ACS, n (%)		2499 (39%)	2,300 (40%)	199 (30%)	0.21	< 0.001
Systolic blood pressure at admin, mmHg	2.00	128 (115, 142)	129 (116, 143)	125 (113, 139)	0.16	< 0.001
Diastolic blood pressure at admin, mmHg	2.10	70 (62, 81)	71 (62, 81)	68 (60, 78)	0.27	< 0.001
Pulse rate at admin, beats/min	2.50	70 (62, 81)	70 (62, 81)	70 (62, 80)	0.03	0.639
Hypertension, n (%)		5522 (86%)	4,947 (85%)	575 (87%)	− 0.03	0.44
Dyslipidemia, n (%)		5189 (80%)	4,678 (81%)	511 (77%)	0.1	0.017
Diabetes, n (%)		2807 (44%)	2,516 (43%)	291 (44%)	− 0.01	0.864
CKD, n (%)		2824 (44%)	2,471 (43%)	353 (53%)	− 0.21	< 0.001
Hemodialysis, n (%)		414 (6.4%)	375 (6.5%)	39 (5.9%)	0.03	0.546
Prior heart failure hospitalization, n (%)		443 (6.9%)	382 (6.6%)	61 (9.2%)	− 0.1	0.013
Prior myocardial infarction, n (%)		1036 (16%)	937 (16%)	99 (15%)	0.04	0.394
Prior stroke, n (%)		752 (12%)	649 (11%)	103 (16%)	− 0.13	0.001
Peripheral artery disease, n (%)		556 (8.6%)	495 (8.6%)	61 (9.2%)	− 0.02	0.582
Atrial fibrillation, n (%)		295 (4.6%)	261 (4.5%)	34 (5.1%)	− 0.03	0.476
Prior PCI, n (%)		1325 (21%)	1,177 (20%)	148 (22%)	− 0.05	0.239
Previous CABG, n (%)		354 (5.5%)	315 (5.4%)	39 (5.9%)	− 0.02	0.645
Culprit lesion						
LMT lesion, n (%)		425 (6.6%)	363 (6.3%)	62 (9.3%)	− 0.11	0.003
LAD lesion, n (%)		4347 (67%)	3915 (68%)	432 (65%)	0.05	0.177
LCX lesion, n (%)		2553 (40%)	2293 (40%)	260 (39%)	0.01	0.816
RCA lesion, n (%)		3165 (49%)	2869 (50%)	296 (45%)	0.1	0.015
Multivessel disease, n (%)		3120 (48%)	2815 (49%)	305 (46%)	0.05	0.186
Laboratory						
BNP, pg/mL	8.20	67 (27, 193)	65 (26, 192)	81 (37, 202)	0.06	< 0.001
Hgb, g/dL	0.30	12.78 (11.43, 13.89)	12.83 (11.50, 13.94)	12.34 (10.96, 13.35)	0.29	< 0.001
Platelet count, 10^3	0.30	198 (165, 239)	199 (166, 239)	194 (158, 238)	0.04	0.016
Creatinine, mg/dL	8.20	0.90 (0.75, 1.13)	0.89 (0.75, 1.12)	0.94 (0.78, 1.16)	0.03	0.002
HbA1c, %	11.40	6.10 (5.70, 6.80)	6.10 (5.70, 6.80)	6.15 (5.70, 6.78)	0.06	0.256
Ejection Fraction, %	14.30	61 (51, 68)	61 (51, 67)	61 (52, 68)	− 0.06	0.185
Medication						
Aspirin, n (%)		5864 (91%)	5262 (91%)	602 (91%)	0.01	0.822
P2Y12 inhibitor, n (%)		5782 (90%)	5209 (90%)	573 (86%)	0.12	0.003
Prasugrel, n (%)		1529 (24%)	1378 (24%)	151 (23%)	0.03	0.539
DAPT, n (%)		5514 (85%)	4969 (86%)	545 (82%)	0.1	0.009
Anticoagulant, n (%)		728 (11%)	653 (11%)	75 (11%)	0	0.993
DOAC, n (%)		315 (4.9%)	276 (4.8%)	39 (5.9%)	-0.05	0.211
Warfarin, n (%)		429 (6.7%)	392 (6.8%)	37 (5.6%)	0.05	0.239
ACEi/ARB, n (%)		4232 (66%)	3850 (67%)	382 (58%)	0.19	< 0.001
β-blocker, n (%)		3950 (61%)	3,609 (62%)	341 (51%)	0.22	< 0.001
Statin, n (%)		5216 (81%)	4761 (82%)	455 (69%)	0.32	< 0.001
Ezetimibe, n (%)		341 (5.3%)	323 (5.6%)	18 (2.7%)	0.14	0.002
PCSK-9 inhibitor, n (%)		4 (< 0.1%)	4 (< 0.1%)	0 (0%)	0.04	> 0.999
PPI, n (%)		5279 (82%)	4793 (83%)	486 (73%)	0.23	< 0.001

Data are mean ± SD, or *n* (%). Data for this parameter were measured at admission

BMI indicates body mass index, *ACS* acute coronary syndrome, *CKD* chronic kidney disease, *PCI* percutaneous coronary intervention, *CABG* coronary artery bypass grafting, *RCA* right coronary artery, *LAD* left anterior descending artery, *LCX* left circumflex artery, *LMT* left main trunk, *BNP* B-type natriuretic peptide, *EF* ejection fraction, *DAPT* dual antiplatelet therapy, *DOAC* direct oral anticoagulants, *ACEi/ARB* angiotensin coverting enzyme inhibitor/ Angiotensin II receptor blocker, *PCSK-9* proprotein convertase subtilisin/kexin type 9, *PPI* proton pump inhibitor

### Primary outcome

**Fig. 2:**
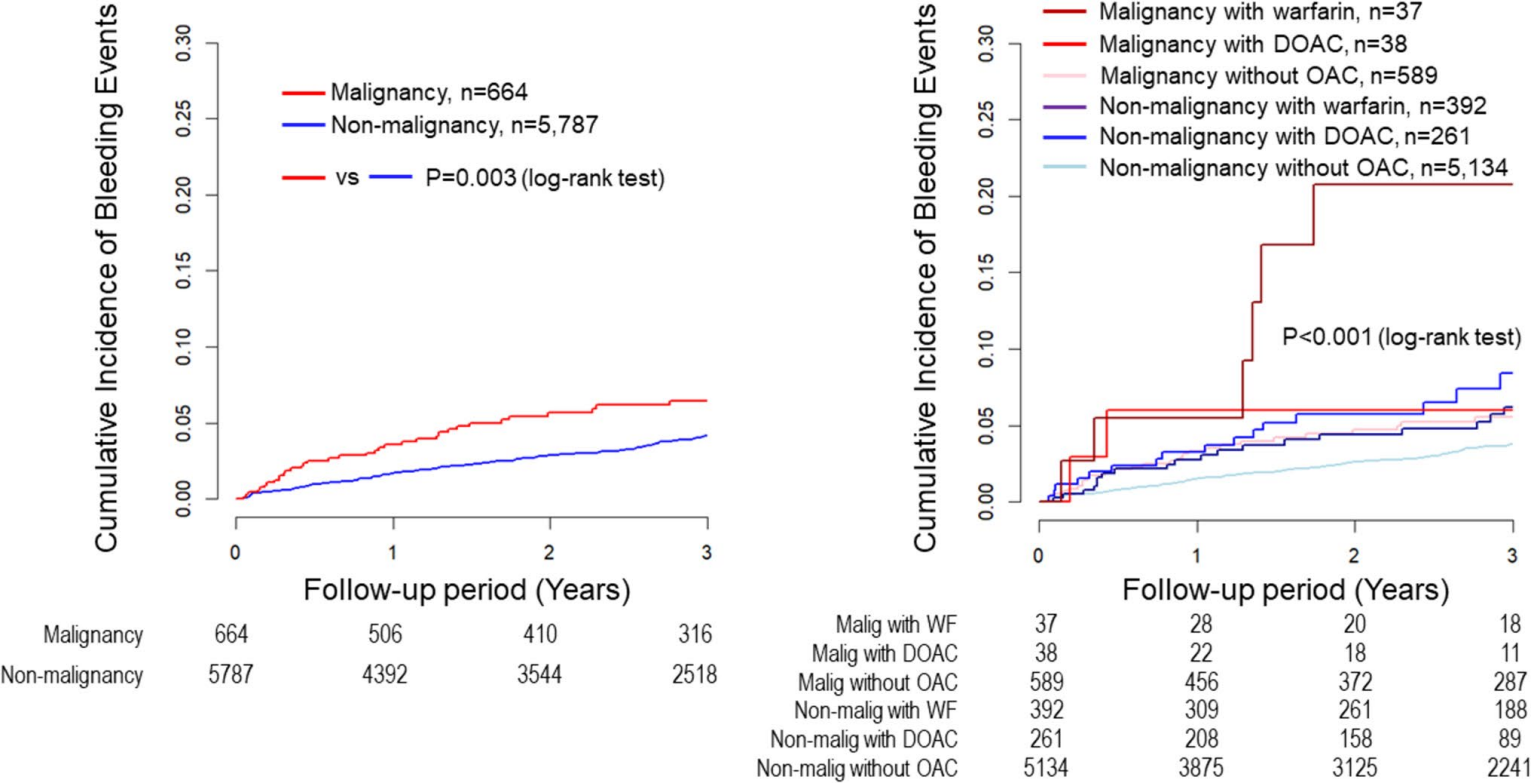
Kaplan–Meier bleeding event estimates for non-malignancy vs. malignancy groups and six malignancy/OAC subgroups. Kaplan–Meier survival curves showed a higher rate of bleeding events in the Malignancy group than in No Malignancy group (p = 0.003) and significant variability among the six groups (p < 0.001). *OAC*, oral anticoagulants, *DOAC* direct oral anticoagulants

**Fig. 3:**
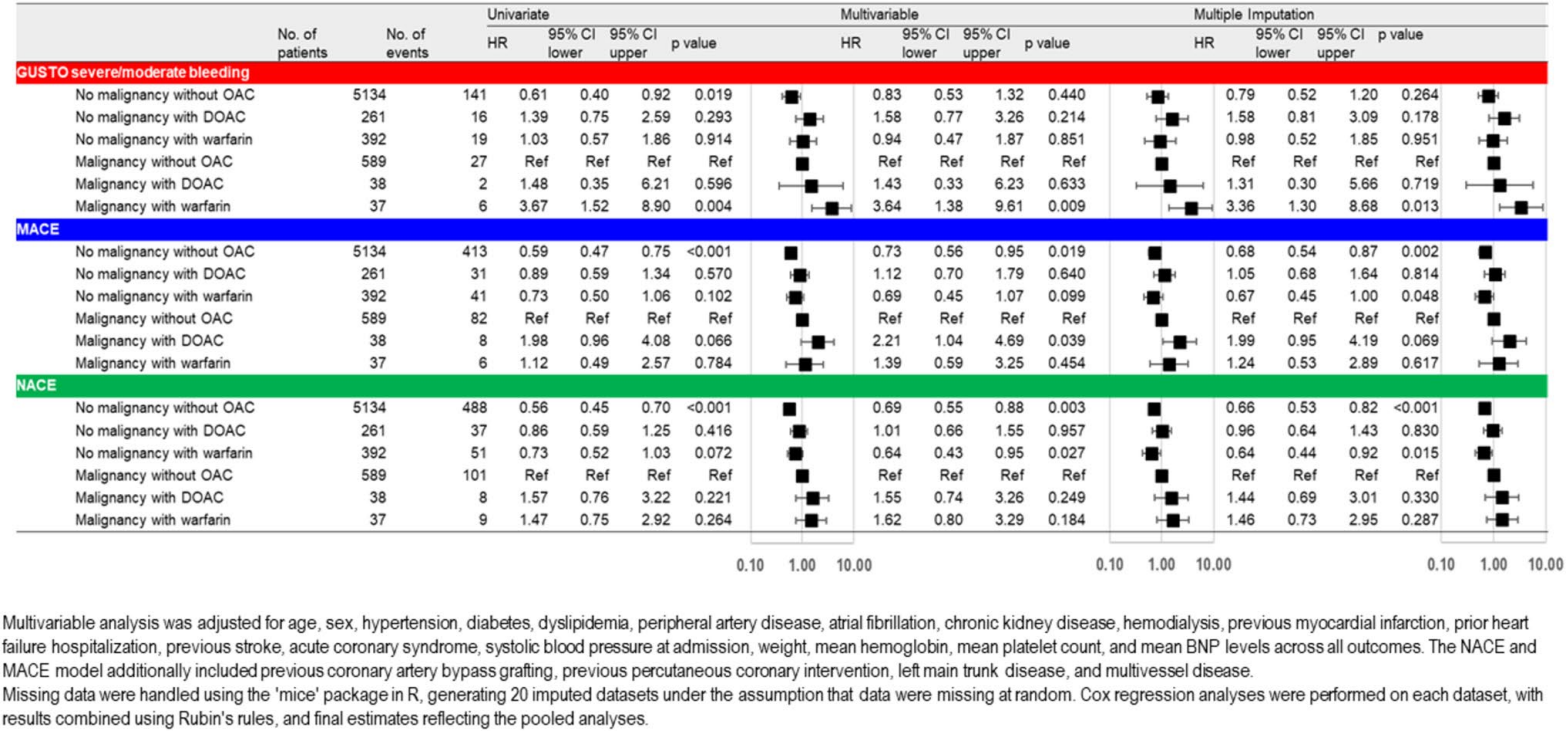
Cox proportional hazard regression for clinical outcomes In the multivariable Cox regression analysis, compared to the Malignancy without OAC group, only the Malignancy with WF group demonstrated a significantly higher incidence of bleeding events and similar findings were observed in the multiple imputation analysis. For the second primary outcome, regarding MACE, multiple imputation analysis revealed that the No Malignancy without OAC group and the No Malignancy with WF group had a lower event rate compared to the Malignancy without OAC group. Similarly, for NACE, the No Malignancy without AC group and the No Malignancy with WF group demonstrated a significantly lower incidence of events in the multiple imputation analysis. *GUSTO* Global Use of Streptokinase and t-PA for Occluded Coronary Arteries, *OAC* oral anticoagulants, *DOAC* direct oral anticoagulants, *HR* indicates hazard ratio, *CI* confidence interval, *HF* heart failure, *BNP* B-type natriuretic peptide, *MACE* major adverse cardiac events, *NACE* net adverse clinical events

During the 3-year follow-up, 213 (3.3%) patients experienced major bleeding events, with 176 in the No Malignancy group and 35 in the Malignancy group. Kaplan–Meier survival curves showed a higher rate of bleeding events in the Malignancy group than that in the No Malignancy group (p = 0.003) and significant differences among the six subgroups (p < 0.001) (Fig. 2). Multivariable Cox regression analysis revealed that, compared to the Malignancy without OAC group, only the Malignancy with WF group demonstrated a significantly higher risk of bleeding events (HR, 3.64; 95% CI 1.38–9.61; p = 0.009), and consistent with the result of the multiple imputation analysis (HR 3.36; 95% CI 1.30–8.68; p = 0.013). However, no significant differences in bleeding risk were observed among the other 4 groups (Fig. 3).

Gastrointestinal bleeding was significantly more frequent in the Malignancy group compared to the No Malignancy group (p < 0.001), while other types of bleeding (intracranial, p value = 0.795; puncture site, p value > 0.99; trauma, p value > 0.99; other bleeding, p value = 0.228) showed no significant differences (**Supplemental **Fig. 1). Among the six groups, significant variability was observed for intracranial bleeding (p = 0.005), gastrointestinal bleeding (p = 0.001), and other bleeding types (p = 0.034), with the Malignancy with WF group exhibiting the highest rate of gastrointestinal bleeding (**Supplemental **Fig. 2).

### Secondary outcomes

**Fig. 4:**
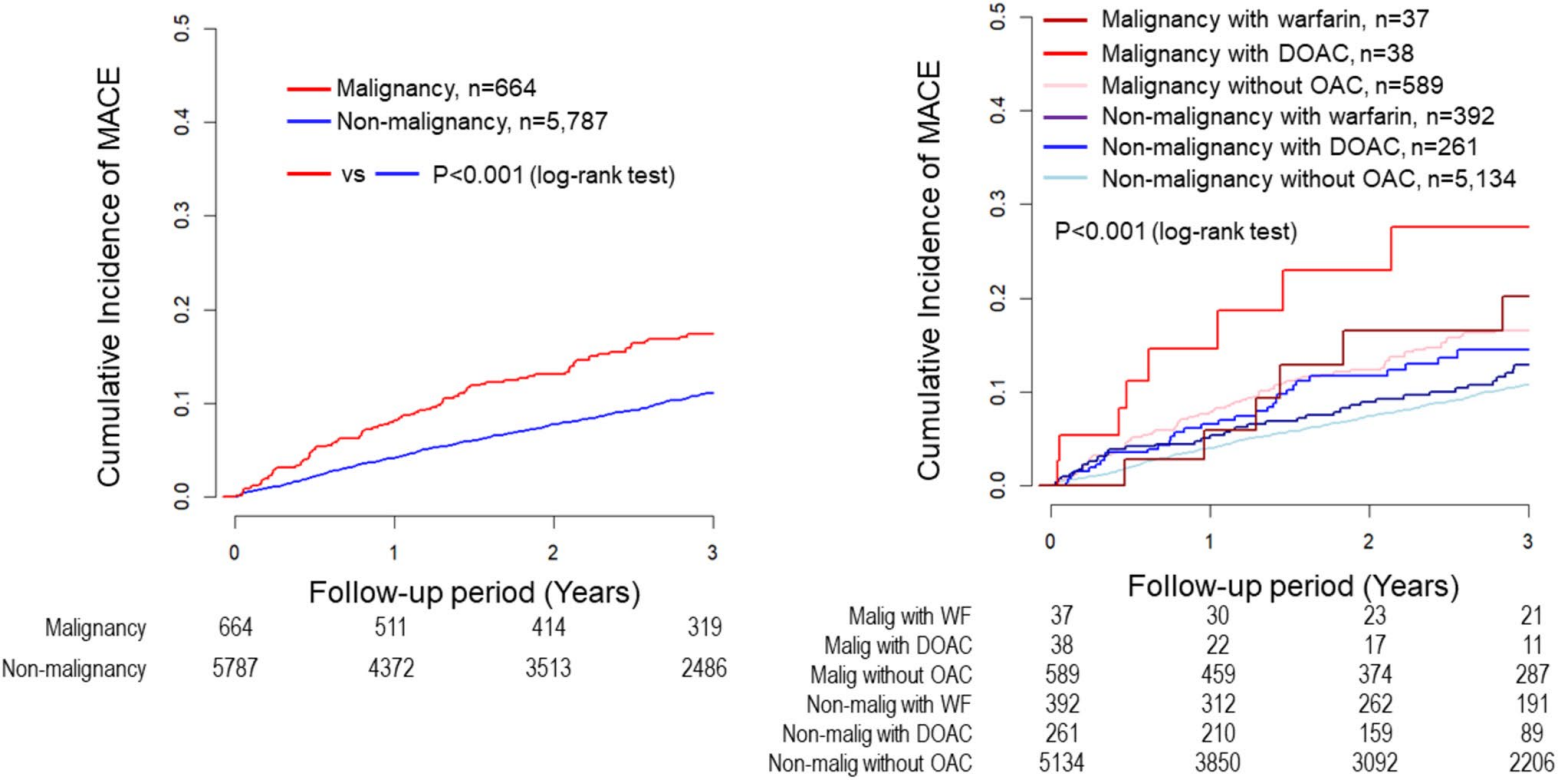
Kaplan–Meier MACE estimates for non-malignancy vs. malignancy groups and six malignancy/OAC therapy subgroups. Kaplan–Meier survival curves showed a higher rate of MACE in the Malignancy group than in No Malignancy group and significant variability among the six groups. *MACE* major adverse cardiac events, *OAC* oral anticoagulants, *DOAC* direct oral anticoagulants

After PCI, 581 (9.0%) patients experienced MACE during the 3-year follow-up, with 485 in the No Malignancy group and 96 in the Malignancy group. Kaplan–Meier survival curves showed a higher rate of MACE in the Malignancy group than in No Malignancy group (p < 0.001) and significant differences among the six subgroups (p < 0.001) (Fig. 4). In the multivariable Cox regression analysis, compared to the Malignancy without OAC group, the No malignancy without OAC group had significantly lower MACE risk (HR, 0.73; 95% CI, 0.56–0.95; p = 0.019), while the Malignancy with DOAC group had a higher MACE risk (HR 2.21; 95% CI 1.04–4.69; p = 0.039) (Fig. 3). Multiple imputation analysis confirmed that both the No Malignancy without OAC group and the No Malignancy with WF group had lower MACE rates compared to the Malignancy without OAC group (HR, 0.68; 95% CI, 0.54–0.87; p = 0.002, HR, 0.67; 95% CI, 0.45–1.00; p = 0.048) (Fig. 3).

**Fig. 5:**
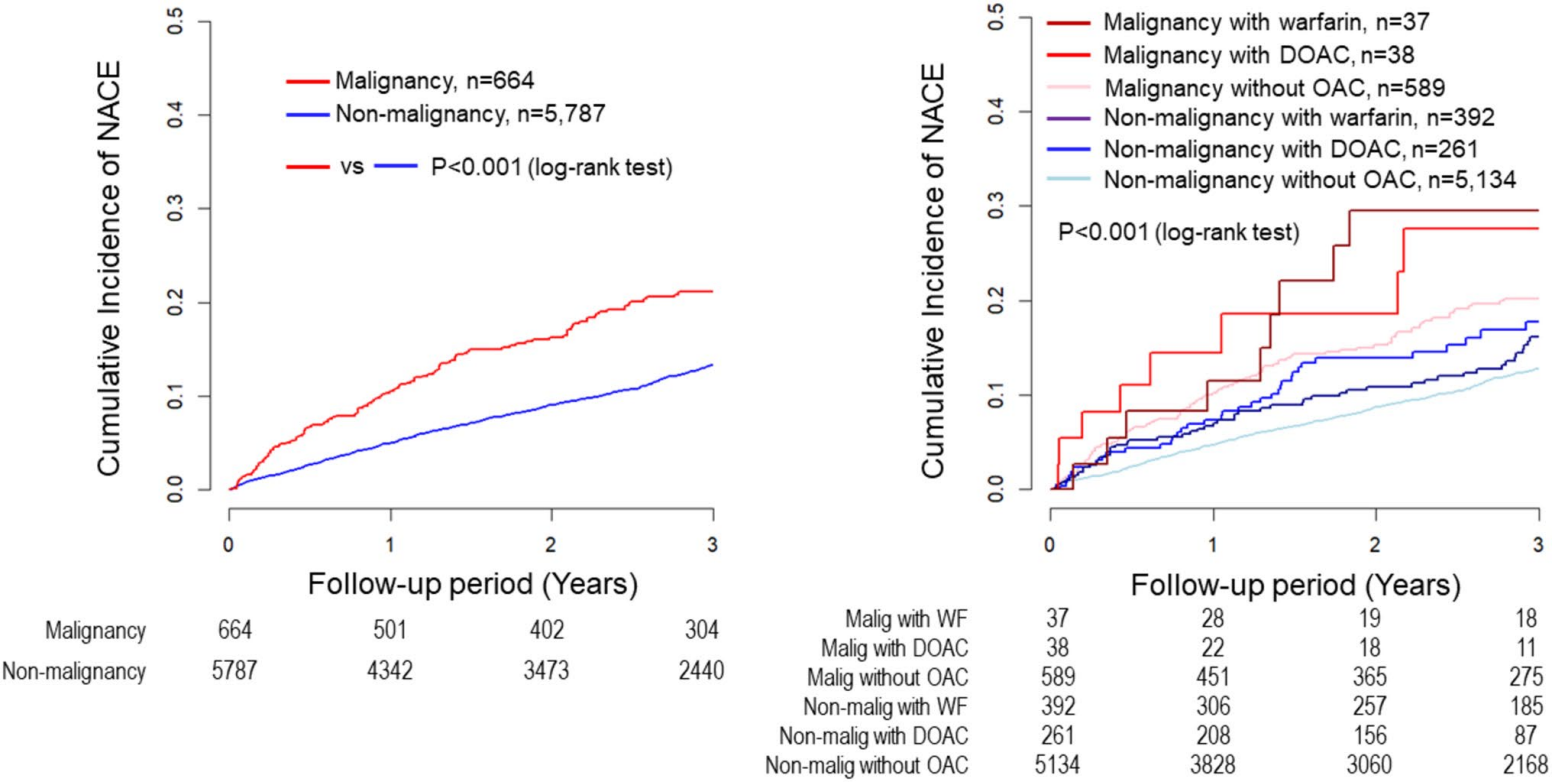
Kaplan–Meier NACE estimates for non-malignancy vs. malignancy groups and six malignancy/OAC therapy subgroups. Kaplan–Meier survival curves showed a higher rate of NACE in the Malignancy group than in No Malignancy group and significant variability among the six groups. *NACE* net adverse clinical events, *OAC* oral anticoagulants, *DOAC* direct oral anticoagulants

NACE occured in 694 (10.7%) patients during the 3-year follow-up. Kaplan–Meier survival curves showed a higher rate of NACE in the Malignancy group than in No Malignancy group (p < 0.001), with significant differences among the six subgroups (p < 0.001) (Fig. 5). Multivariable Cox regression analysis indicated that the No Malignancy without OAC group and the No Malignancy with WF group had significantly lower NACE rates (HR, 0.69; 95% CI, 0.55–0.88; p = 0.003, and HR, 0.64; 95% CI, 0.43–0.95; p = 0.027). These findings were supported by the multiple imputation analysis (HR, 0.66; 95% CI, 0.53–0.82; p < 0.001, HR, 0.64; 95% CI, 0.44–0.92; p = 0.015) (Fig. 3).

## Discussion

This large-scale, multicenter, observational cohort study of 6451 patients who underwent PCI during a 3-year follow-up period investigated the association between malignancy and bleeding events. Given the limited reports directly comparing DOAC and WF for anticoagulant therapy in patients with malignancy after PCI, the findings of this study provide valuable insights. Regarding the primary outcome, the present study showed that a higher incidence of bleeding events in the Malignancy group compared to the No Malignancy group, supporting the inclusion of malignancy as a HBR factor in both the ARC-HBR and Japan-HBR criteria. Moreover, patients with malignancy receiving warfarin after PCI experienced a higher incidence of bleeding events, highlighting the elevated bleeding risk associated with warfarin in this population (**Graphical abstract**).

The overall incidence of bleeding events was 3.3% in our cohort, consistent with the 1.6% to 7.3% range reported in previous studies [20–22]. In addition, the prevalence of malignancy in patients undergoing PCI was 10.3%, aligning with previously reported rates of 3.8% to 13.3% [5], [23–25]. Furthermore, gastrointestinal bleeding was more frequent than intracranial bleeding, a finding consistent with previous studies on post-PCI complications [26].

Further investigation is required to explore the reasons why cancer patients undergoing PCI, particularly those treated with warfarin, are more prone to bleeding events. The increased bleeding risk in cancer patients treated with warfarin may be attributed to multiple. factors. Tumor-related bleeding, hepatic dysfunction, medications, radiation therapy, malnutrition, thrombocytopenia, and disseminated intravascular coagulation (DIC) are potential contributors. Patients with malignancy had a higher incidence of bleeding events when treated with OAC compared to those without malignancy, with similar findings observed in studies specifically focused on warfarin [27, 28]. Furthermore, this population has a significantly higher rate of transfusions during the perioperative period of PCI and experience more frequent readmissions for bleeding compared to those without malignancy following PCI for AMI [29, 30]. These study findings clearly demonstrate that a malignant condition leads to an increased susceptibility to bleeding.

Warfarin metabolism relies on hepatic cytochromes P450 (CYP) enzymes, and its clearance may be impaired by hepatic dysfunction, which can be exacerbated by cancer or congestive hepatopathy following PCI [31]. This impaired clearance can lead to enhanced anticoagulant effects. Additionally, conditions such as hypoalbuminemia, common in cancer patients due to malnutrition or nephrotic syndrome, increase the proportion of unbound warfarin, further enhancing its anticoagulant effect and elevating risk of bleeding [32, 33].

Regarding anticancer agents, 5-fluorouracil, molecular targeted therapies such as gefitinib and imatinib, and anticancer drugs like tamoxifen used in the treatment of breast cancer have been reported to enhance the effects of warfarin by several reason such as inhibiting CYP enzymes [27], [34]. Optimal INR control in warfarin therapy for cancer patients is challenging in the context of cancer treatments, leading to an increased risk of bleeding and other significant cardiovascular adverse events [35]. In light of these risks, warfarin administration in patients with malignancy is associated with an increased incidence of bleeding events following PCI. The selection of anticoagulant therapy was left to the discretion of the attending physicians at each institution. In general, DOAC were favored in patients with non-valvular atrial fibrillation, preserved renal function, and no contraindications such as high-risk gastrointestinal lesions or potential drug interactions with anticancer agents. Warfarin was more often chosen for patients with advanced renal dysfunction, mechanical heart valves, or unstable coagulation profiles requiring close INR monitoring. These real-world prescribing patterns may have introduced indication confounding, as baseline characteristics and clinical complexity likely differed between the DOAC and warfarin groups. Although we adjusted for potential confounders and applied multiple imputation for missing data, unmeasured confounding cannot be entirely excluded.

In terms of the secondary outcomes, multivariable Cox regression analysis demonstrated a significantly higher incidence of MACE in the Malignancy with DOAC group compared to the Malignancy without OAC group, while no significant difference was observed in the Malignancy with WF group. Several potential explanations may account for this finding. First, some malignancies are generally associated with a hypercoagulable state through mechanisms such as tissue factor expression, platelet activation, and the release of inflammatory cytokines [36]. These biological processes can increase thrombotic risk regardless of the anticoagulant used. In this study, however, detailed information, including cancer type, stage, activity, and treatment status, was not available. Moreover, the choice of anticoagulant type was at the discretion of attending physicians, introducing the possibility of indication confounding. In clinical practice, it is also plausible that DOAC were preferentially prescribed to patients with more active malignancy or higher thrombotic risk, as supported by prior trials investigating DOAC use in cancer patients at elevated risk for thromboembolic events [37, 38]. In this context, it is conceivable that some patients in the DOAC group had a history of cancer-associated thrombosis, such as venous thromboembolism, and were prescribed DOAC accordingly. This may have contributed to the higher incidence of thrombotic events observed in the malignancy with DOAC group. In addition, variations in the duration and modification of post-discharge antiplatelet therapy may have influenced the observed differences in MACE incidence. Second, the discrepancy may be attributed to the small sample sizes in the malignancy groups treated with DOAC and WF (n = 38 and n = 37, respectively), which may have introduced that statistical variability and led to unstable estimates. Therefore, further prospective studies with larger cohorts and more detailed oncologic and pharmacologic data are needed to clarify the cardiovascular safety profile of DOAC in cancer patients undergoing PCI. Although current guidelines recommend DOAC over WF for AF, the benefits of DOAC in cancer patients, particularly in relation to thromboembolic events such as ischemic stroke, have produced inconsistent results across studies [39, 40]. Given the limited number of studies comparing OAC therapies in cancer patients following PCI, as in this study, further research with larger sample sizes is warranted to better assess MACE and NACE outcomes in this population.

This study has several limitations. First, because this was a retrospective study, selection bias and unmeasured confounding factors, such as the type, progression, treatment history of the malignancy, the duration and modification of antiplatelet therapy, and transfusion history, may have influenced the results. In particular, the CLIDAS database did not consistently capture detailed information on the continuation or discontinuation of dual antiplatelet therapy (DAPT) after hospital discharge. As previous trials (e.g., WOEST, PIONEER AF-PCI, and RE-DUAL PCI) have demonstrated that prolonged triple therapy increases bleeding risk, the lack of granular antiplatelet data is a significant limitation and may have affected the observed clinical outcomes [41–43]. To reduce selection bias, this study included all patients from the seven hospitals during the study period. Second, owing to the nature of the database, the definition of malignancy was based on a history of prior malignancy, which differs from the active malignancy definition used in the ARC-HBR and Japan-HBR criteria. Despite this broader definition, it is unlikely that the bleeding risks in our malignancy group were overestimated. Third, the CLIDAS database includes data from the Japanese Diagnosis Procedure Combination System, which limits the traceability following hospital transfers. As a result, some bleeding events may have been underestimated due to incomplete follow-up for patients treated at other hospitals post-PCI.

## Conclusion

Patients with malignancy receiving warfarin were associated with a higher risk of bleeding events following PCI. Although DOAC use was not associated with an increased bleeding risk, a significantly higher incidence of MACE was observed in the DOAC group compared to the non-OAC group among patients with malignancy. This discrepancy should be interpreted with caution, particularly in light of the limited number of patients in the DOAC group. Further research with larger sample sizes is needed to clarify the long-term safety profile of DOAC compared to warfarin in this population.

### Clinical perspective

Competencies in Medical Knowledge: Malignancy patients undergoing PCI face elevated bleeding risks, especially with warfarin. DOACs may represent a preferable alternative to warfarin with regard to bleeding risk, potentially reducing bleeding complications. Utilizing a large multicenter dataset strengthens insights into anticoagulant choice in high-risk PCI patients.

Translational Outlook: The findings advocate for preferential use of DOAC over warfarin in cancer patients requiring PCI, potentially decreasing bleeding events. This approach could guide future anticoagulation strategies in oncology and cardiology, addressing rising PCI procedures among aging cancer populations with high bleeding risks.

## Supplementary Information

Below is the link to the electronic supplementary material.

Supplementary file1 (DOCX 19 KB)

Supplementary file2 (TIF 82 KB)

Supplementary file3 (TIF 102 KB)

## Data Availability

The data underlying this article will be shared on reasonable request to the corresponding author.
