# Supplementary material for: Long-term bleeding events post-percutaneous coronary intervention in patients with malignancy with and without anticoagulant therapy
Source: Cardiovasc Interv Ther. 2025 Jun 9;40(4):796–806. doi: 10.1007/s12928-025-01151-4 (PMC12431914; doi:10.1007/s12928-025-01151-4)
Supplement: Supplementary file 1 — Supplementary file1 (DOCX 19 KB) [file 12928_2025_1151_MOESM1_ESM.docx]

**Supplement for Long-term Bleeding Events after Percutaneous Coronary Intervention in Patients with Malignancy with and without Anticoagulant Therapy**

Otsuka Y, et al.

**CONTENTS**

**Supplemental Figure 1. Types of bleeding events in patients with and without malignancy**

Gastrointestinal bleeding was significantly more frequent in the Malignancy group compared to the No Malignancy group, while no significant differences were noted in other bleeding events.

GUSTO, Global Use of Streptokinase and t-PA for Occluded Coronary Arteries

**Supplemental Figure 2. Bleeding event types across six malignancy/OAC groups classified by DOAC or warfarin therapy.**The Malignancy with WF group exhibited the highest proportion of gastrointestinal bleeding.

OAC, oral anticoagulants; DOAC, direct oral anticoagulants
