# Supplementary figures and images for: Long-term bleeding events post-percutaneous coronary intervention in patients with malignancy with and without anticoagulant therapy
Source: Cardiovasc Interv Ther. 2025 Jun 9;40(4):796–806. doi: 10.1007/s12928-025-01151-4 (PMC12431914; doi:10.1007/s12928-025-01151-4)

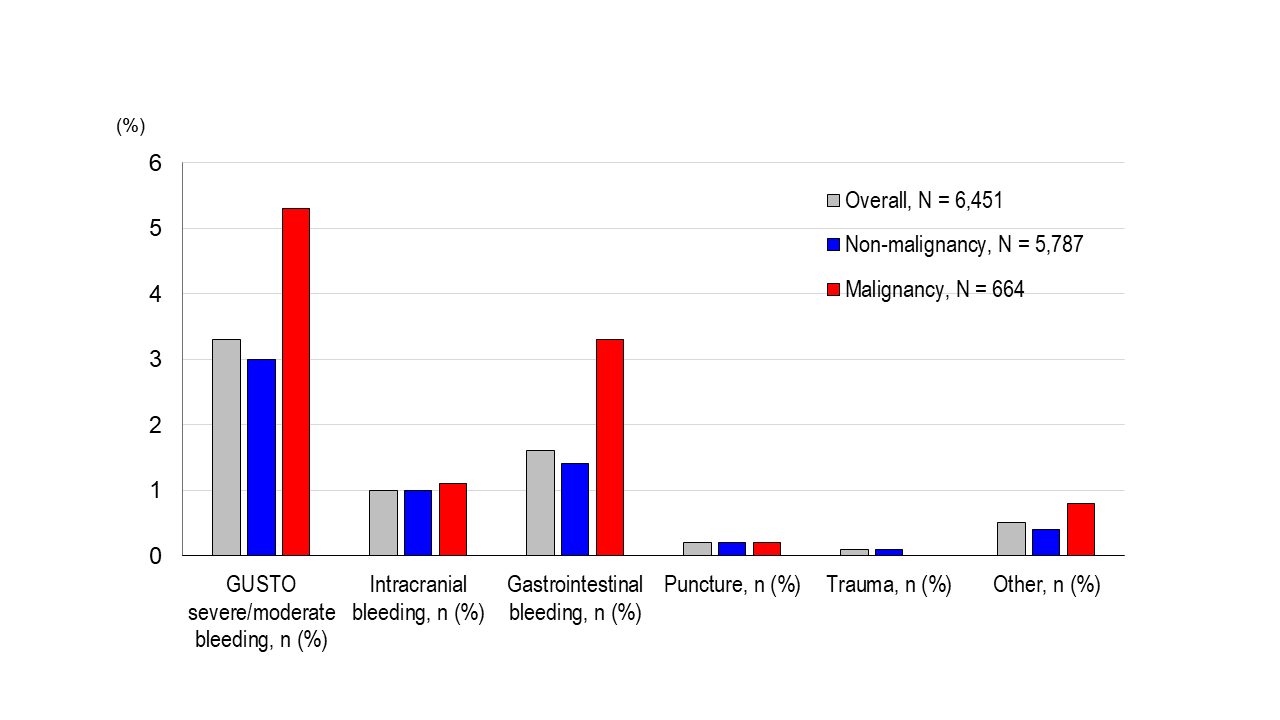

Supplement: Supplementary file 2 — Supplementary file2 (TIF 82 KB) [file 12928_2025_1151_MOESM2_ESM.tif]

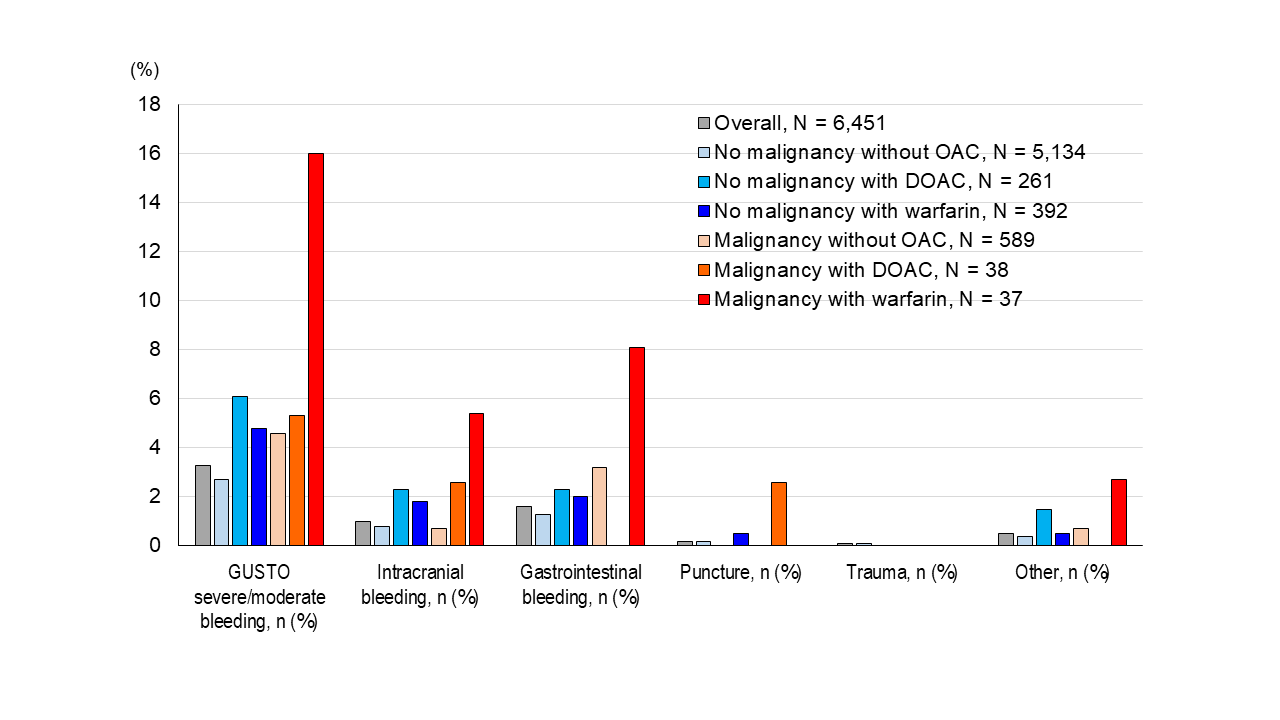

Supplement: Supplementary file 3 — Supplementary file3 (TIF 102 KB) [file 12928_2025_1151_MOESM3_ESM.tif]
